# Early maturing *Bt* cotton requires more potassium fertilizer under water deficiency to augment seed-cotton yield but not lint quality

**DOI:** 10.1038/s41598-019-43563-2

**Published:** 2019-05-14

**Authors:** Ahmad Naeem Shahzad, Muhammad Rizwan, Malik Ghulam Asghar, Muhammad Kamran Qureshi, Syed Asad Hussain Bukhari, Aysha Kiran, Abdul Wakeel

**Affiliations:** 1 Department of Agronomy, Bahauddin Zakariya University, Multan, 60800 Pakistan; 2 Department of Plant Breeding and Genetics, Bahauddin Zakariya University, Multan, 60800 Pakistan; 3 Department of Botany, University of Agriculture, Faisalabad, 38000 Pakistan; 4 Institute of Soil and Environmental Sciences, University of Agriculture, Faisalabad, 38000 Pakistan

**Keywords:** Plant physiology, Abiotic

## Abstract

Exhaustive crops such as cotton require potassium (K) in copious amounts as compared to other crops. High yielding cultivars in cotton-wheat cropping system, have further increased its demand in cotton growing areas of Pakistan. As cotton is grown in arid and semiarid areas, therefore often prone to water deficiency. The reproductive growth particularly flowering and boll setting are highly sensitive to low soil water potentials, where enough K supply can play a vital role. In this two-year field studies, three cultivars (early, mid and late maturing) were cultivated at two K fertilizer levels 100, 200 kg K ha^−1^ along with control with no K fertilizer application at two irrigation levels. In first irrigation level, water was applied as per full irrigation schedule, while in water deficit irrigation water was applied at deficit irrigation schedule started after flowering till harvesting. It has been revealed that K application has impact on boll setting as well as seed cotton yield, however early and mid-maturing cultivars are more responsive to K fertilization. Furthermore, irrigation level had significant impact against K fertilization and relatively better response was observed in deficit irrigation as compared to full irrigation. Nevertheless, fiber quality parameters were unaffected by K fertilization. Considering the best benefit cost ratio under water deficiency, it is concluded that 100 kg K_2_O ha^−1^ should be applied at the time of seed bed preparation for economical seed-cotton yield of early maturing *Bt* cotton.

## Introduction

Increase in agricultural production is a great challenge that is affected by various biotic and abiotic stresses which can be mitigated to certain extent through nutrient management. Potassium (K) is one of the essential macronutrients required by plants that is involved in numerous physiological and biochemical processes affecting plant growth and development. Potassium has great contribution in biotic or abiotic stresses amelioration and its deficiency enhance the severity of these stresses^1^. Maintenance of charge balance across the cell membranes, osmotic adjustment, water relations, stomatal regulation, enzyme activation and resistance against biotic and abiotic stresses are among established functions of K in plants^2^. Therefore, adequate availability of K is pre-requisite for optimal growth and reproductive success of plants. Although most of the soils have enough K, however continuous mining without addition of K fertilizer has lowered the available K levels in the soils. Pakistani soils are specifically becoming deficient in plant available K because of intensive cropping without application of K fertilizers^3^.

Importance of K fertilization has increased for exhaustive crops like cotton which require K in plentiful amounts. Despite high requirements, K fertilizer use in cotton production in Pakistan is very limited causing further K mining from the soils and lowering K levels in soils^3,4^. High yielding cultivars and continuous cultivation of cotton, in cotton-wheat cropping system, have exhausted the soils of cotton region of Pakistan too much. Furthermore, cotton is grown in arid- and semiarid areas which are often prone to lack of water. The reproductive growth particularly flowering and boll setting are highly sensitive to low soil water potentials. Potassium, being involved in stomatal regulation and cell water relations, plays significant role in conferring drought resistance to plants under field conditions^5^. Use of K fertilizer can minimize the effects of drought stress and drought sensitive varieties are more responsive to K fertilizers than drought tolerant ones^6^. Drought stress leads to stomatal closure, resulting in poor intercellular carbon dioxide concentration, which reduces the net photosynthesis (Pn), and transport of photosynthates towards reproductive parts. The application of K fertilizer significantly ameliorated the drought stress by increasing Pn, accumulation of biomass and it’s partitioning^7^. Water deficiency decreases plant height, leaf area index, yield of seed cotton, lint length, uniformity, micronaire and lint resistance^8,9^.

High yields in the early maturing modern cultivars are the factors responsible for appearance of K deficiency symptoms in cotton^10^, and lack of extensive root system may be a reason for K deficiency. However, variations in yield response of different cotton cultivars to K availability does exist and genotypic differences in yield are more related to K uptake rather than K utilization under K deficient conditions. It has been observed that early planting cotton cultivars bear more number of bolls with more boll weight as compared to mid and late planting cultivars^11^. As application of K fertilizer in cotton leads to increased boll weight, K fertilization is more important for early planting modern *Bt* cotton cultivars. Potassium fertilization to early planting cotton cultivars results in increased boll weight and fruit bearing branches, while deficiency of K-fertilization can cause delayed maturation of bolls^12,13^. K deficiency also led to reduced leaf area, number of bolls, weight of seed cotton per boll and lint percentage^14^. Deficiency of K affects reproductive growth of cotton, by reducing translocation of sugars, causing a decline in lint and boll weight, which ultimately reduces the yield^15^.

The present study was conducted to investigate the agronomic response of *Bt* cotton cultivars differing in maturing periods to different K applications comparing water-sufficient and water-deficient field conditions.

## Methods

**Figure 1:**
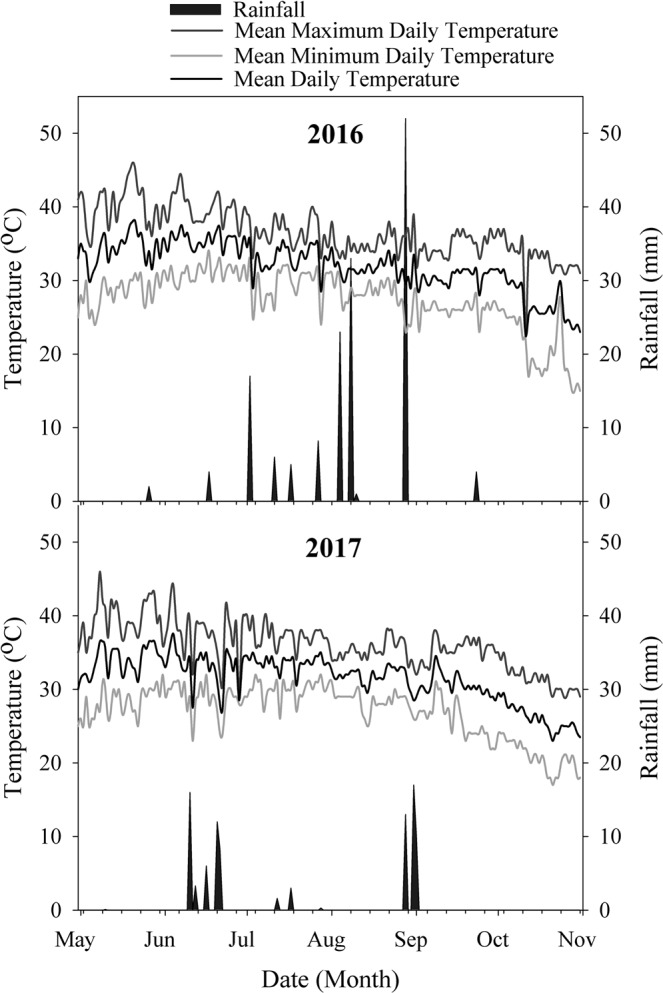
Meteorological data for 2016 and 2017.

A two-year field study (2016 and 2017) was conducted at the research area of Department of Agronomy, Bahauddin Zakariya University Multan. The site is described as semi-arid region with an average annual rainfall of 150–200 mm. The meteorological measurements i.e. daily rainfall, daily mean temperature, daily mean maximum and minimum temperatures are described in Fig. 1. The soil type was silty-clay in texture, with 8.4 pH and 3.39 dS m^−1^ EC. The top soil contains 0.63% organic matter, 0.03% total nitrogen, 9.11 mg kg^−1^ soil olsen phosphorus and ammonium acetate exchangeable K was 140 mg kg^−1^ soil.

Three *Bt* upland cotton (*Gossypium hirsutum* L.) cultivars [early-maturing: *Bt* CIM-602 (V_ear_), mid-maturing: *Bt* CIM-616 (V_mid_) and late-maturing: *Bt* CIM-622 (V_late_)], having different maturity times, were developed at Central Cotton Research Institute (CCRI), Multan, Pakistan. These varieties were selected based upon their maturity time from the germplasm of CCRI. All three cultivars have a semi-erect growth habit and are adapted to the local conditions of Multan region of the Punjab province. A Randomized Complete Block Design (RCBD) experiment with split-split plot arrangement and three replicates was laid out. Irrigation levels (full irrigation and deficit irrigation) were kept in main plot, K fertilizer rates (No K fertilizer, 100 and 200 kg K ha^−1^) in sub-plots and three cultivars were placed in sub-sub plots.

Before planting, a presoaking irrigating was applied to the field, and seed bed was prepared after attaining the soil moisture at field capacity. Cotton cultivars were planted on April 30, 2016 and May 01, 2017 in individual plots of size 2.25 m × 6 m. Seeds were planted manually on 75 cm apart ridges with plant to plant spacing of 30 cm. Gaps, if any, were filled on 30 days after sowing to maintain a uniform plant density (44,444 plants ha^−1^) across all treatments. Based on current recommendations for cotton, 120 kg P_2_O_5_ ha^−1^ was applied as di-ammonium phosphate at the time of seed bed preparation. Nitrogen (200 kg N ha^−1^) was applied as urea in three equal splits i.e. 1/3 at sowing, 1/3 at bud formation and 1/3 at peak flowering stage. Potassium fertilizer in the form of potassium chloride was applied to the respective treatment plots at the time of seed bed preparation. To schedule irrigation, tensiometers were installed in the field at depths of 45 cm and 66 cm. Tensiometers were calibrated at two irrigation thresholds i.e. 75% field capacity for full irrigation treatment (25% soil water depletion) and 50% field capacity (50% soil water depletion) for deficit irrigation treatment. All plots were irrigated with equal amount of irrigation water when soil moisture reached 75% or 50% field capacity for full and deficit irrigation treatments, respectively. Deficit irrigation was started after the initiation of flowering and continued until crop reached boll cracking stage. After considering rainfall, full irrigation treatments received 1094 mm and 1005 mm water during 2016 and 2017 (9 irrigations each year), respectively, while deficit irrigation treatments received 788 and 699 mm water during 2016 and 2017 (6 irrigations each year), respectively.

Five plants from each plot were randomly selected and tagged for measurements of plant height and bolls per plot at 90, 105, 120 and 135 DAS, respectively. Fifty opened bolls were selected from each plot to record the average boll weight. Seed-cotton was picked three times and yield was calculated in kg ha^−1^. To determine the influence of irrigation and K and cultivar on fiber quality, ginning out turn (%), staple length (mm), uniformity index (%), micronaire, and fiber strength G/tex 1/8” were measured.

Ginning out turn was calculated as described below.

$${\rm{Ginning}}\,\mathrm{out}\,\,{\rm{turn}}\,( \% )=\frac{{\rm{weight}}\,{\rm{of}}\,{\rm{lint}}}{{\rm{total}}\,{\rm{weight}}\,\mathrm{of}\,\,{\rm{seed}}\,{\rm{cotton}}}\times 100$$

Potassium concentration was measured in leaves subtending cotton bolls after digesting the sample with perchloric and nitric acids and K was determined using flamephotometer. The leaf samples were collected before last picking of seed cotton.

### Statistical analysis

Data were subjected to analysis of variance using Statistix 8.1 for split-split plot design^16^. Orthogonal contrasts were used to test the linear and quadratic responses of traits to potassium fertilizer. Regression lines were determined using SPSS 18.0. Treatment means, where appropriate, were compared using LSD test at P ≤ 0.05.

## Results

The three cultivars were characterized based upon the traits such as days from sowing to first flower (DSFF), days from sowing to first boll (DSFB) and node number to first sympodial branch (NNFSB). The cultivars V_ear_, V_mid_ and V_late_ recorded the DSFF as 46.7 ± 0.67, 47.7 ± 0.88 and 51.0 ± 0.58, respectively. Whereas, the DSFB recorded for V_ear_, V_mid_ and V_late_ were 92.0 ± 0.58, 95.5 ± 0.76 and 100.3 ± 0.88, respectively. The values of NNFSB recorded for V_ear_, V_mid_ and V_late_ were 7.6 ± 0.06, 7.8 ± 0.09, and 8.1 ± 0.08, respectively.

**Figure 2:**
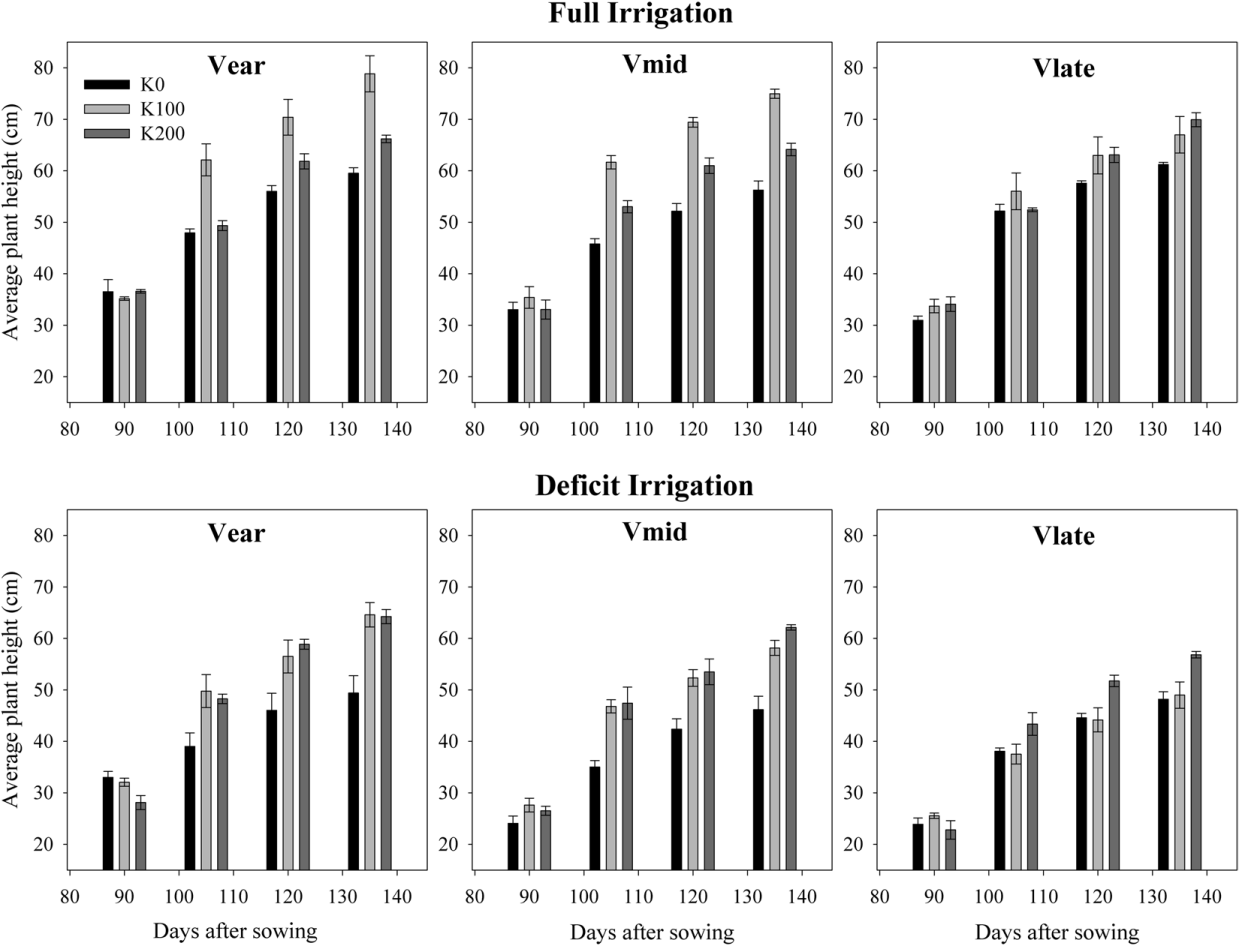
Plant height as affected by irrigation levels, K rates and cultivar at 90, 105, 120, and 135 days after sowing. Data are means with error bars indicating SD (n = 3). V_ear_, V_mid_ and V_late_ represent early, medium and late maturing cotton cultivars, respectively.

Water stress due to deficit irrigation led to reduction in plant height of all cultivars. Moderate application of K (100 kg K ha^−1^) significantly increased plant height under both full and deficit irrigation (Fig. 2). In V_ear_ under full irrigation, plant height linearly increased with K fertilizer rate (No K fertilizer to 100 kg K ha^−1^) at 90, 105, 120 and 130 DAS, but higher dose of K fertilizer (200 kg K ha^−1^) had no significant effect on plant height as compared to control (No K fertilizer). In deficit irrigation maximum plant height of V_ear_ was observed at 100 kg ha^−1^ K rate which was significantly higher than control. However, plant height at 200 kg K ha^−1^ application was statistically at par with that of 100 kg^−1^ K ha^−1^. Equivalent results on plant height were obtained for V_mid_. In V_late_ at 90 DAS the plants did not respond to K fertilizer but at 105 and 120 DAS, a slight increase in plant height was recorded under full irrigation. After 135 DAS plant height of V_late_ was considerably higher than control at 200 kg K ha^−1^ fertilizer rate. Under water stress (deficit irrigation) there was linear increase in plant height of V_late_ with K application i.e. maximum plant height was observed in 200 kg ha^−1^ K application. Overall response of V_ear_ and V_mid_ in full irrigation was maximum at moderate K fertilizer rate (100 kg ha^−1^), while in deficit irrigation increment in K rate linearly increased plant height of all cultivars.

**Figure 3:**
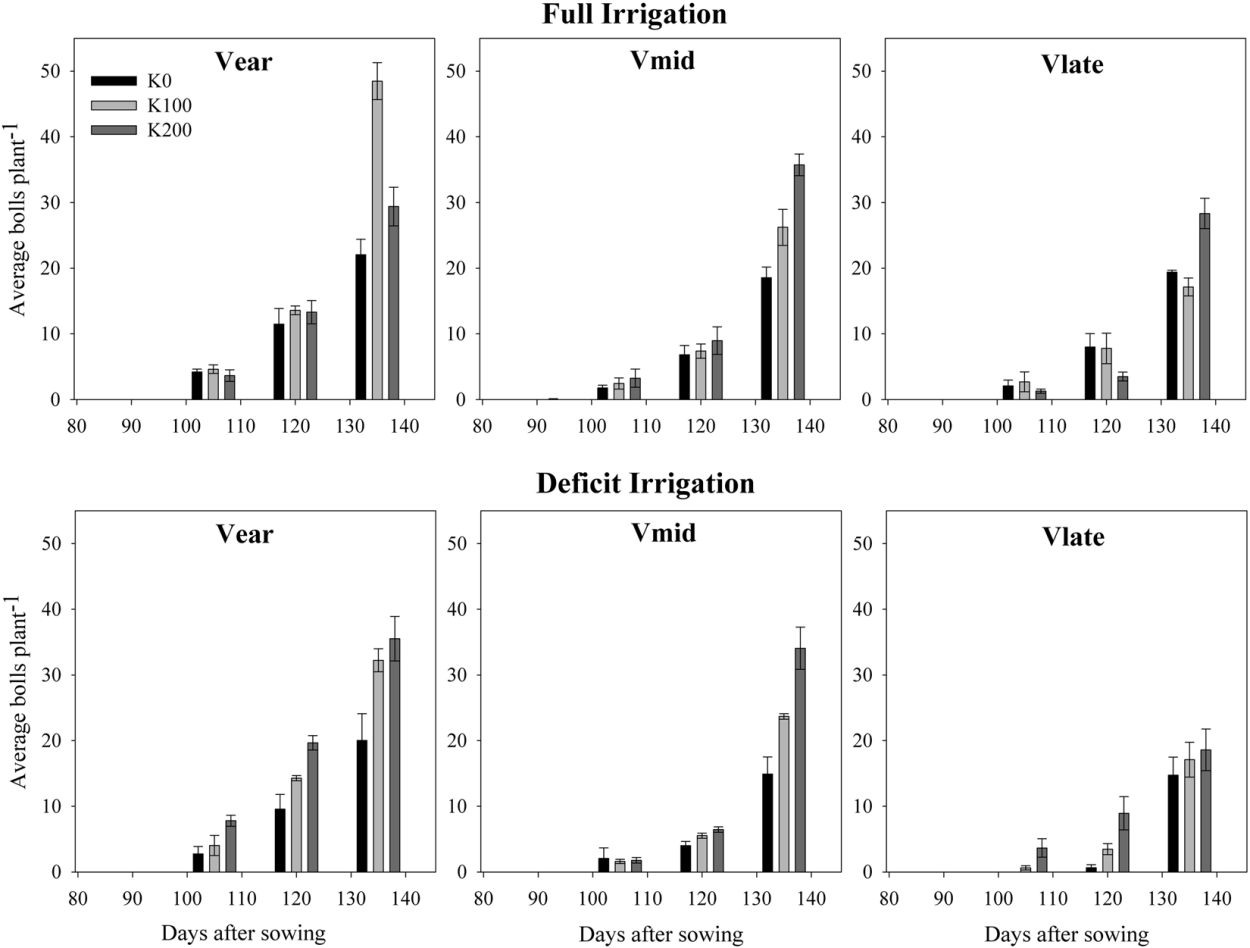
Bolls per plant as affected by irrigation levels, potassium rates and cultivars at 90, 105, 120, and 135 days after sowing. Data are means with error bars indicating SD (n = 3). V_ear_, V_mid_ and V_late_ represent early, medium and late maturing cotton cultivars, respectively.

Average number of bolls per plant was reduced under deficit irrigation in comparison with full irrigation in all cultivars (Fig. 3). Maximum number of bolls per plant was observed in V_ear_ in both full and deficit irrigation. With increase in K fertilizer rate (from 0 to 200 kg K ha^−1^), the number of bolls per plant varied in full irrigation but increased linearly in all the three cultivars under deficit irrigation. Number of bolls per plant of V_mid_ also responded linearly to K fertilizer rate in full irrigation.

**Figure 4:**
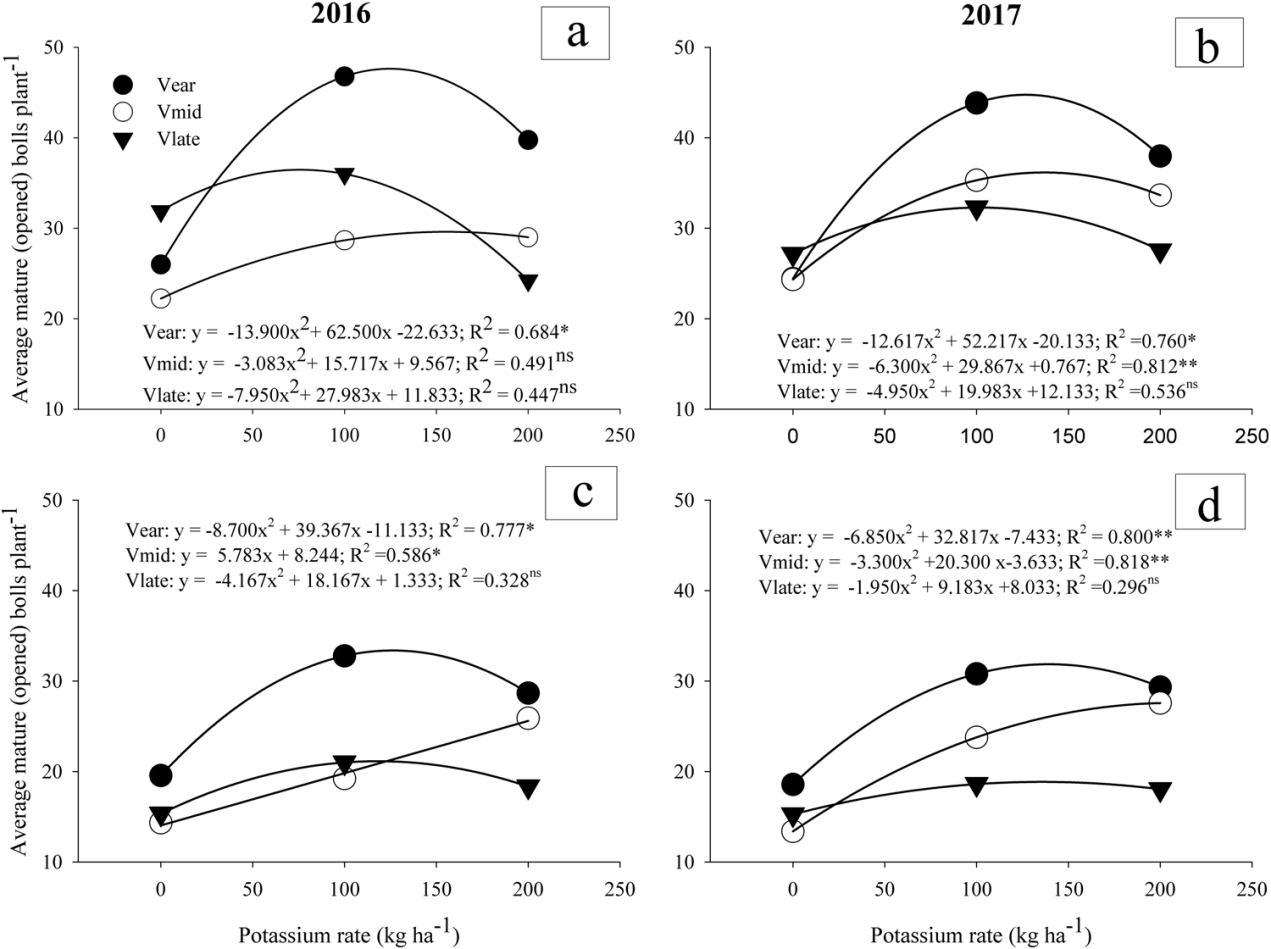
Mature (opened) bolls of early, mid and late-maturing cotton cultivars as affected by three potassium rates (0, 100 and 200 kg ha^−1^) and two irrigation levels (full and deficit) in 2016 and 2017. Data are means with error bars indicating SD (n = 3).

**Figure 5:**
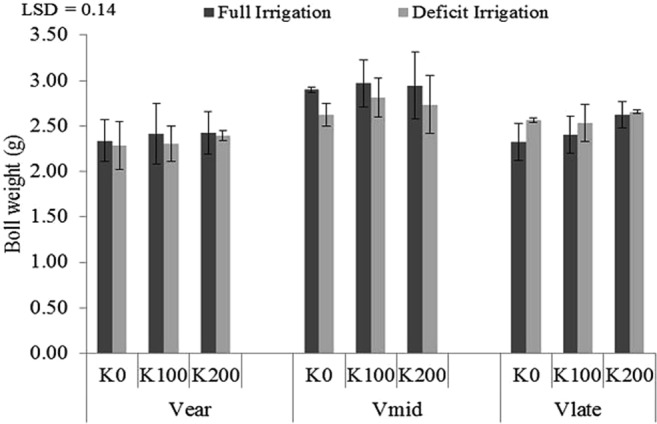
Boll weights of early, mid and late-maturing cotton cultivars as affected by three potassium rates (0, 100 and 200 kg ha^−1^) and two irrigation levels (full and deficit). Data are means with error bars indicating SD (n = 3).

All the three cultivars had quadratic relation with K fertilizer levels under both full and deficit irrigations except V_mid_ which was linear with slight slope during first year under both full and deficit irrigation. Analysis of variance (ANOVA) exhibited significant effects of K application under full and deficit irrigation in all the three cotton cultivars. Deficit irrigation had detrimental effects on average mature (opened) bolls (Fig. 4). Under full irrigation, number of mature (opened) bolls in V_late_ were more as compared to V_ear_ and V_mid_ at no application of K while K application at both levels (100 and 200 kg ha^−1^) lead to quadratic decrease in number of bolls in V_late_. Significant decrease in boll number was observed in V_late_ at high application of K fertilizer (200 kg ha^−1^). V_ear_ was responsive to K fertilizer application during both years in full irrigation. Maximum number of bolls was observed in V_ear_ at 100 kg ha^−1^ during both years. At no application of K, no difference in number of bolls was observed in V_ear_ and V_mid_. The response of all varieties towards K application was quadratic in full and deficit irrigation. V_late_ was non-significant in full and deficit irrigation system. Regression analysis showed V_ear_ was very responsive to K application of 100 kg K ha^−1^ in deficit irrigation with perfectly quadratic curve having value of R^2^ 0.78 in 2016 and 0.88 in 2017. Average number of opened bolls was significantly more in V_ear_ under water deficit condition. The cultivar V_mid_ exhibited significantly higher boll weights than both V_ear_ and V_late_ (Fig. 5). However, K application and irrigation treatments had no significant impact on boll weights of all cultivars.

**Figure 6:**
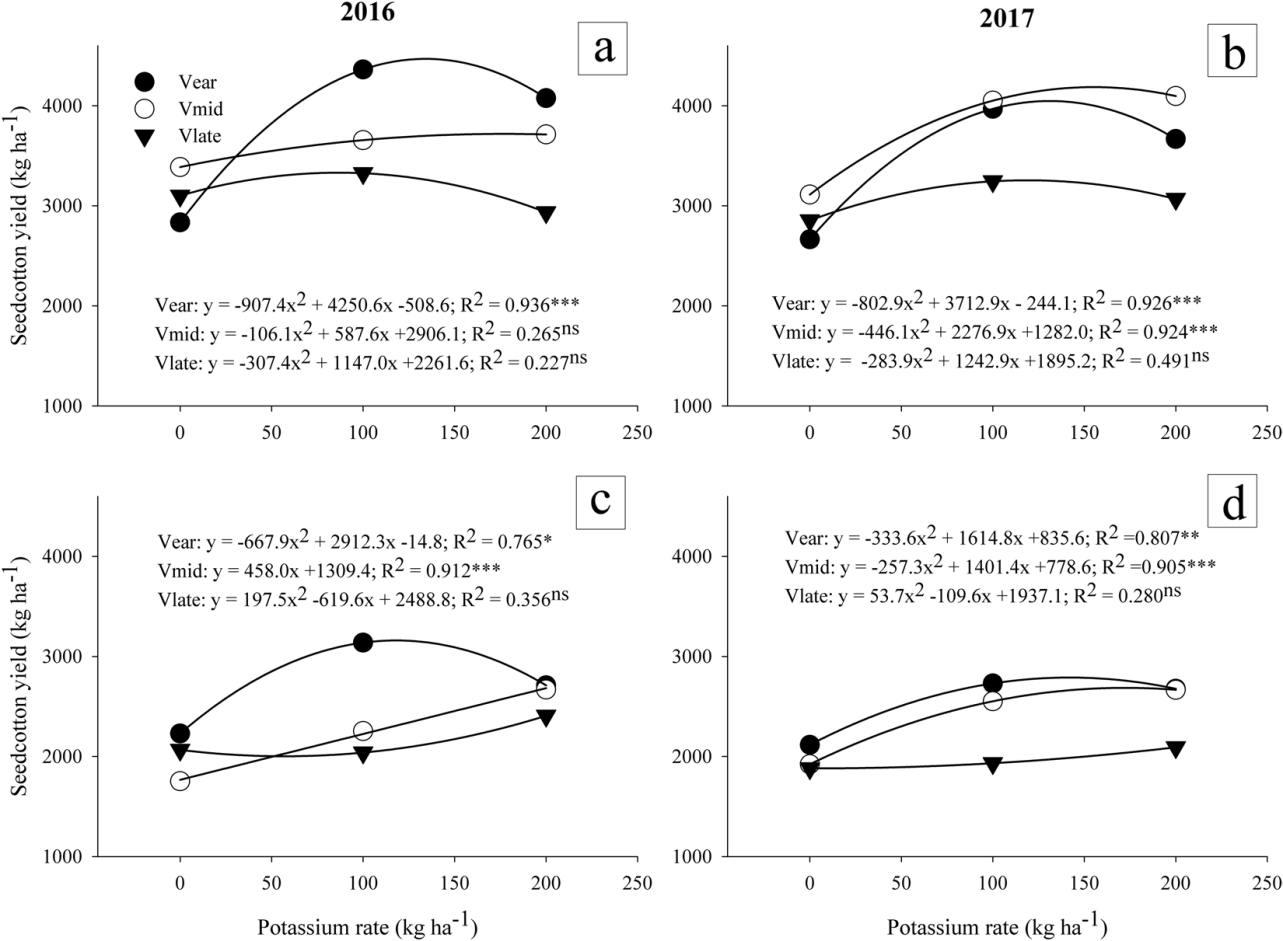
Seed cotton yield of early, mid and late-maturing cotton cultivars as affected by three potassium rates (0, 100 and 200 kg ha^−1^) and two irrigation levels (full and deficit) in 2016 and 2017. Data are means with error bars indicating SD (n = 3).

Deficit irrigation had a negative effect on all the three cultivars. A highly significant reduction in yield was observed under deficit irrigation. The effect of K on seed cotton yield differed significantly between cultivars and irrigation schedules (Fig. 6). In full irrigation, a quadratic regression was observed between seed cotton yield and levels of K, except for V_mid_, which was less quadratic during 2016 (Fig. 6a,b). Statistical analysis showed that interaction of cultivars with K application was highly significant (P < 0.001) during both years (Table 1). In both irrigation schedules (full and deficit irrigation) a significant quadratic relation was observed between seed cotton yield and levels of K in all cultivars. A strong quadratic relation of seed cotton yield with K was recorded in V_ear_ during year 2016 under both irrigation regimes i.e. increasing level of K fertilizer form No K fertilizer to 100 kg K ha^−1^ increased the seed cotton yield in both irrigation conditions while for year 2017 relation was slight quadratic. An additional increment of K fertilizer from 100 kg ha^−1^ to 200 kg ha^−1^ resulted in decline of seed cotton yield of V_ear_ under both irrigation levels. In both years under full irrigation increase of K from 100 kg ha^−1^ to 200 kg ha^−1^ slightly increased seed cotton yield of V_mid_ while same increment slightly decreased seed cotton yield in V_late_. In deficit irrigation V_mid_ showed positive linear regression relation in year 2016 (R^2^ = 0.912). Increment in K fertilizer lead to increase in seed cotton yield but in year 2017 V_mid_ was slightly quadratic and overlapped with V_ear_ at 200 kg ha^−1^.

**Table 1:** Summary of the analysis of variance (ANOVA) for the effect of irrigation levels, potassium rates and cultivar on growth, yield and quality attributes of cotton.

Source of variation	I	K	I*K	V	I*V	K*V	I*K*V
Df	1	2	2	2	2	4	4
Bolls plant^−1^ at 90 DAS	ns	ns	ns	ns	ns	ns	ns
Bolls plant^−1^ at 105 DAS	ns	ns	ns	***	ns	ns	ns
Bolls plant^−1^ at 120 DAS	ns	**	**	***	ns	ns	ns
Bolls plant^−1^ at 135 DAS	ns	***	ns	***	ns	***	**
Plant height at 90 DAS	*	ns	ns	***	*	ns	ns
Plant height at 105 DAS	*	***	***	ns	*	**	ns
Plant height at 120 DAS	***	***	**	*	ns	*	ns
Plant height at 135 DAS	***	***	**	***	*	***	ns
Boll weight (g)	ns	ns	ns	***	ns	ns	ns
Mature (opened) bolls plant^−1^ (2016)	*	**	ns	***	ns	**	ns
Mature (opened) bolls plant^−1^ (2017)	*	***	ns	***	ns	**	ns
Seed cotton yield (2016)	***	***	ns	***	*	***	*
Seed cotton yield (2017)	**	***	*	***	***	***	ns
Leaf potassium	ns	***	ns	ns	ns	ns	ns
Fiber strength	ns	*	ns	***	ns	*	ns
Micronaire	ns	*	ns	***	**	***	***
Staple length	ns	*	ns	***	ns	***	ns
Uniformity index	ns	ns	ns	ns	ns	ns	ns
Ginning out turn	ns	ns	ns	ns	ns	ns	ns

ns: No significant effects.

*Significant effect at P < 0.05 level.

**Significant effect at P < 0.01 level.

***Significant effect at P < 0.001 level.

As the K fertilizer level increased response of seed cotton yield was observed which was reduced at further increment in K fertilizer rate. At 0 kg K ha^−1^ application no difference in seed cotton yield of all three cultivars was observed in full and deficit irrigation but there was slight increase in seed cotton yield of V_mid_ in full irrigation system. Regression line was linear in V_mid_ during both years with slight slope i.e. increased application of K fertilizer increased seed cotton yield in 2016 but next year curve of V_mid_ was also quadratic as others. During both years V_ear_ showed minimum seed cotton yield without K fertilizer application but at 100 kg K ha^−1^ seed cotton yield was maximum as compared to other cultivars under full irrigation, V_mid_ showed maximum seed cotton yield in year 2017 at all levels of K fertilizer as compared to other cultivars. Seed cotton yield of V_late_ was lower at all levels of K fertilization as compared to other cultivars under full irrigation system. Under deficit irrigation seed cotton yield of V_ear_ was more at 0 kg K ha^−1^ and was maximum in V_ear_ at 100 kg ha^−1^ as compared to V_mid_ and V_late_. There was quadratic decrease in seed cotton yield in response of K fertilization. Under deficit irrigation seed cotton yield of V_mid_ overlapped with V_ear_ at 200 kg K ha^−1^ application. The number of bolls per plant was lower in V_mid_ as compared to V_ear_ in both irrigation levels but more seed cotton yield was observed in V_mid_ (Fig. 3).

**Figure 7:**
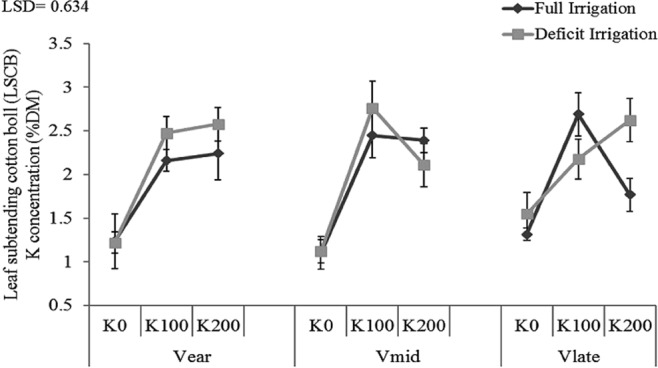
K concentration in leaf subtending cotton boll (LSCB) of early, mid and late-maturing cotton cultivars as affected by three potassium rates (0, 100 and 200 kg ha^−1^) and two irrigation levels (full and deficit) in 2016 and 2017. Data are means with error bars indicating SD (n = 3).

Increased K fertilizer application in deficit irrigation showed increasing trend in K accumulation in leaf subtending cotton boll (LSCB) in all cultivars with exception of V_mid_ which showed reduction of K accumulation at 200 kg ha^−1^. Under full irrigation, all cultivars showed variations from one another. Concentration of K in LSCB was low in V_mid_ at all K fertilizer rates with similar trend in deficit irrigation (Fig. 7). In V_mid_ concentration of K in LSCB was maximum at 100 kg ha^−1^ but further increment in K fertilization (200 kg ha^−1^) slightly decreased its concentration. In V_late_ a linear relation of K concentration in LSCB with K rate was observed under deficit irrigation.

Under full irrigation no significant effect of K application was observed on fiber quality of cotton in all cultivars and increment of K fertilizer (no K fertilizer to 200 kg K ha^−1^) had no effect on fiber strength, micronaire and staple length (Table 2). However, under deficit irrigation fiber quality was slightly better than in full irrigation. Overall no significant difference in fiber quality was recorded among cultivars under both irrigation regimes at all K fertilizer levels. In deficit irrigation improvement of quality was slightly correlated with K fertilizer rate i.e., staple length in V_ear_ and V_mid_ increased with minor value.

**Table 2:** Ameliorative role of potassium supplementation under drought stress on fiber length, micronaire and staple length of 3 cotton cultivars.

	Potassium rate (kg ha^−1^)	Fiber strength		Micronaire		Staple length	
		Full Irrigation	Deficit Irrigation	Full Irrigation	Deficit Irrigation	Full Irrigation	Deficit Irrigation
V_ear_	0	28.6	29.2	3.5	3.3	26.2	27.1
	100	30.6	29.8	3.0	3.1	28.8	27.7
	200	30.7	30.3	3.1	3.3	28.4	28.2
V_mid_	0	29.0	27.4	3.5	3.8	26.9	25.0
	100	30.3	29.0	3.2	3.5	28.0	27.0
	200	29.6	28.7	3.6	3.4	27.5	27.4
V_late_	0	28.3	29.2	3.4	3.0	26.2	27.5
	100	28.2	28.2	3.6	3.3	26.3	25.5
	200	27.8	28.0	3.5	3.5	26.2	25.9
LSD		1.436		0.222		1.197	

## Discussion

Cotton crop has a great significance as a cash crop of Pakistan. Pakistani soils are rich in total K as parent material, however available K is being depleted and more than 40% soils are deficient in plant available K^3^. Being an exhaustive crop, cultivation of cotton continuously on the same field accelerated the K depletion. Furthermore, climate change is affecting water reservoirs and water scarcity in dry areas leads to increase in drought spells. Optimum K nutrition can minimize the deleterious effects of drought on crop plants. *Bt*-transgenic cultivars of cotton are more sensitive to K deficiency than non-*Bt* conventional cultivars due to its high yield potential and long duration^17^. In the present study, we observed that deficit irrigation significantly influenced seed cotton yield. A quadratic relation of V_ear_ effectively described that it is very responsive to K application which might be due to the fact that our soils are becoming deficient in K. Nevertheless, differential response of cultivars to K fertilization is an interesting outcome of this study.

Although all cultivars of cotton used in this study were responsive to soil-applied K, however more responsiveness of early maturing cultivars to K fertilization may be due to short duration and bearing more number of bolls. It shows that requirement of K fertilizers differs with respect to maturity period and growth habit of cotton^18^. Lower K fertilizer application response to cotton growth was more economical as compared to higher dose showing that soils are not severely K deficient and can support growth to certain extent. On the other hand, water deficiency has been reported to have clear effect on plant height, leaf area index and dry matter accumulation^8^. Potassium is proved to be most important plant macro-nutrient for cotton with regard to water relations as it directly influences physiological processes and regulation of plant developmental attributes^1^. Under deficit irrigation plant height was increased in K treated plots than control in these studies (Fig. 2) as drought sensitive cultivars are more responsive to K fertilizer than the resistant cultivars^6^. Increase in total dry mass in crops is due to adequate amounts of K under drought stress which might be attributable to higher rates of photosynthesis and stomatal regulation. Additionally, K plays critical role in translocation of photoassimilates for root growth. Increase in root surface area enhances water uptake under appropriate K supply^1^.

Water availability at flowering onwards has great significance. Water shortage at this stage leads to decrease in boll setting with ultimate reduction in fiber and seed cotton yield. The current results showed that K application not only improved number of bolls under full irrigation but also had a great contribution in boll setting under deficit irrigation. Perhaps this is due the fact that K plays significant role in plants’ physiological processes such as stomatal conductance, osmoregulation, activation of enzymes, synthesis of proteins, phloem transport and energy transfer^19^. Potassium deficiency leads to decrease in stomatal conductance and gases exchange that result in photosynthesis depression. It is also evident that K deficiency may deteriorate chlorophyll ultrastructure^20,21^ and also causes reduction in chlorophyll contents^22^ that ultimately disturbs photochemical reactions and photosynthetic activities.

Reduced number of bolls per plant under water deficit conditions is due to more requirement of photo-assimilates during flowering and boll setting as reduced water availability leads to reduced partitioning of photosynthates^23^. Potassium plays role in water balancing thus K fertilization improves boll setting specially at boll formation stage. The increase in boll number may be due to higher concentration of K in leaf subtending cotton boll (LSCB) as number of bolls has direct relation with yield^24^. Higher K in LSCB is closely associated with mechanisms of osmoregulation, maintenance of water status and reduced water loss from leaf surface for adaptation in drought-stressed condition^7^. Economical yields were achieved when K was applied at the rate of 100 kg K ha^−1^ to early maturing cotton cultivar (Table 3).

**Table 3:** Economic analysis of K fertilization to three differentially maturing cotton cultivars under full and deficit irrigation conditions.

Treatments			Total Expenditure (USD ha^−1^)		Gross Income (USD ha^−1^)		Net Income (USD ha^−1^)		Benefit cost ratio	
			2016	2017	2016	2017	2016	2017	2016	2017
Full Irrigation	Vear	K0	1123	1152	1844	1815	721	663	1.64	1.58
		K100	1182	1212	2838	2703	1655	1491	2.40	2.23
		K200	1242	1271	2651	2497	1409	1226	2.13	1.96
	Vmid	K0	1123	1152	2203	2119	1081	967	1.96	1.84
		K100	1182	1212	2378	2758	1196	1547	2.01	2.28
		K200	1242	1271	2415	2790	1173	1518	1.94	2.19
	Vlate	K0	1123	1152	2017	1943	894	791	1.80	1.69
		K100	1182	1212	2163	2210	981	998	1.83	1.82
		K200	1242	1271	1909	2089	667	818	1.54	1.64
Deficit Irrigation	Vear	K0	1032	1062	1450	1441	418	380	1.40	1.36
		K100	1092	1121	2041	1859	949	738	1.87	1.66
		K200	1152	1181	1763	1823	612	642	1.53	1.54
	Vmid	K0	1032	1062	1140	1309	108	247	1.10	1.23
		K100	1092	1121	1466	1738	374	616	1.34	1.55
		K200	1152	1181	1736	1816	584	635	1.51	1.54
	Vlate	K0	1032	1062	1344	1281	312	219	1.30	1.21
		K100	1092	1121	1327	1316	234	195	1.21	1.17
		K200	1152	1181	1566	1425	414	244	1.36	1.21

Altering K application rates from 0 to 200 kg ha^−1^ significantly influenced seed cotton yield which was effectively described by quadratic function exception with V_mid_ which was linear in 2016 (Fig. 6). Variation among cultivars in response to K fertilizer may be due to their maturing time. The increment in seed cotton yield may be attributed to increased number of bolls and mature bolls per plant. Potassium fertilization has role in increasing leaf number and size which improve the photosynthetic activity of the plants. More production of photosynthates could accelerate the assimilation rate^25^.

Concentration of K in LSCB plays significant role in boll development. Application of K fertilizer increased biomass of plant and cotton bolls (lint and seed weight). Potassium plays pivotal role in breakdown of starch hence triggers boll filling^14^. The concentration of K in leaf was lower in control than in treated plots. Potassium treatments were characterized by increment in plant height, seed cotton yield and mature bolls per plant. K is found in plants with cytoplasmic concentration of 100–200 mM^26^ while the concentration of K in apoplast may range between 10–200 mM or even up to 500 mM^27^. Decisively, K is involved in most of the sophisticated adaptive mechanisms which evolved in plants to survive under moisture stress. Vital processes affected under moisture stress are changes in the expression of genes, induction of stress proteins along with hormone regulation, osmotic balance, maintenance of membrane stability, generation of antioxidants and rate of carbon fixation rate^28^.

In conclusion K fertilization has clear impact on boll setting and seed cotton yield. Early maturing cultivars seem more responsive to K fertilization. Furthermore, irrigation level had also significant impact against K fertilization and relatively better response was observed under water deficient conditions in early maturing cotton cultivar. Potassium fertilization at 100 kg K_2_O ha^−1^ seems more economical as the best benefit cost ratio was achieved in both water levels. Based on superior agronomic performance and highest benefit cost ratio at 100 kg K_2_O ha^−1^ under both water sufficient and water deficit conditions, the early maturing *Bt* CIM-602 is recommended for sowing.
